# Common Accidents of Every-Day Life

**Published:** 1887-12-17

**Authors:** Robert A. P. Forrester


### THE COMMON ACCIDENTS OF EVERY-DAY LIFE.

By Robert A. P. Forrester, B.A.Dub., M.B. and C.M.Edin.

### ACCIDENT TO A CHILD IN A PERAMBULATOR.

A medical friend of mine related to me a fatal accident which happened to a young child in the streets of London during a very hot summer some two or three years ago, which might have been prevented had the nurse been more attentive to her charge. The little one was out for exercise in a perambulator with its nurse. The latter chose the side of the street exposed to the sun's rays, and seemed more intent on gazing into the shop windows than anything else. During the walk the child had no covering of any kind for its head, and in all probability soon began to feel the effects of the great heat rendered doubly powerful by reflection from the houses and pavement. A gentleman, who happened to look at the child, noticed something peculiar in its appearance, and on examining more carefully found that life was extinct. What had caused this? Undoubtedly, *sunstroke*. Had the nurse been at all careful and anxious she must assuredly have noticed that the little one was not all right, for death from sunstroke does not occur at once. There are premonitory symptoms, and afterwards unconsciousness lasting for longer or shorter periods, so that we may take it for granted she had neglected, and culpably neglected, her duty for some considerable period.

Now, what is sunstroke, and what is the treatment of such an accident?

Sunstroke is a disease of the nervous system induced by heat, sometimes following exposure to the direct rays of the sun. It is apt to occur when the body is exhausted by fatigue in hot weather. The premonitory symptoms are thirst, dryness of the skin, giddiness, blood-shot eyes, then syncope or unconsciousness, with stertorous breathing and convulsions. If during hot weather the head be well protected from the sun's rays, suitable clothing worn, and *no stimulants taken* sunstroke rarely occurs.

It may be confounded with cerebral apoplexy. In apoplexy, however, the pulse is slow, in sunstroke it is quick. The same may be said of the breathing. The pupils are contracted in sunstroke, in apoplexy dilated.

*Treatment.*—Formerly blood-letting used to be employed, but experience has shown that this method generally dose harm. Most of the cases so treated have proved fatal. The proper method to pursue is at once to remove the patient to a cool and shady place, strip him, and douche the head, neck, and chest with cold water. This will reduce the heat of the skin, and if effectually and quickly done will re-establish the breathing. It may be necessary to repeat the process more than once. As soon as the patient has recovered consciousness let him drink freely, because great thirst is complained of, and if vomiting supervene so much the better. In India tamarind water and lime juice are much used. The bowels ought to be moved by enemata. If the skin remain dry and hot, even after the free use of the douche, sudorifics ought to be freely used. Blistering the head (shaven) or the nape of the neck is a good measure when consciousness is not restored for a considerable period.

As the affection appears to depend on shock to the nervous system, and depression of nervous energy, those remedies which rouse the tissues to further action, and support the system, are the most appropriate to administer. As soon, therefore, as the patient can swallow, give stimulants—brandy, wine, and good nourishing food, such as beef-tea, soups, etc.

Another fatal accident occurred to a child in a perambulator in the most simple manner. It happened in this way: The child had a knitted woollen muffler round its neck, one end of which was hanging over the side. This became entangled in the wheel of the carriage, and being wound round and round by the motion, tightened itself on the neck of the poor little sufferer, and so strangled it.

Both the above melancholy accidents might not have occurred at all if only *ordinary* care had been exercised by those in charge of the infants; and as the latter cannot speak for themselves, and thereby draw attention to their wants, it becomes incumbent on the nurses to be doubly vigilant when such a carriage as a perambulator is used for an infant. The employment of a sunshade or a broad-brimmed hat might have saved the child's life in the first instance. Besides, it does no good, but actual harm, to send an infant out during intense heat. Perambulators seem to have been invented only to save nurses trouble. How is it that we hardly ever hear of casualties to invalids in bath chairs?

